# Multi-trait index: selection and recommendation of superior black bean genotypes as new improved varieties

**DOI:** 10.1186/s12870-024-05248-5

**Published:** 2024-06-10

**Authors:** Moisés Ambrósio, Rogério Figueiredo Daher, Raiane Mariani Santos, Josefa Grasiela Silva Santana, Ana Kesia Faria Vidal, Maxwel Rodrigues Nascimento, Cleudiane Lopes Leite, Alexandre Gomes de Souza, Rafael Souza Freitas, Wanessa Francesconi Stida, João Esdras Calaça Farias, Benedito Fernandes de Souza Filho, Leonardo Cunha Melo, Paulo Ricardo dos Santos

**Affiliations:** 1 Center for Agricultural Sciences and Technologies, State Univ. of North Fluminense Darcy Ribeiro, 2000 Alberto Lamego Avenue, Parque Califórnia, 28013-602 Campos dos Goytacazes, RJ Brasil; 2 Empresa de Pesquisa Agropecuária do Estado do Rio de Janeiro, Campos dos Goytacazes – RJ, Av. Francisco Lamêgo, 134 -, Jardim Carioca, Brasil; 3 Empresa Brasileira de Pesquisa Agropecuária - Embrapa Arroz e Feijão, Rodovia GO-462 Km 12 Zona Rural C.P 179 - Santo Antonio De Goias, Goiás, GO Brasil; 4 INSTITUTO FEDERAL DE EDUCAÇÃO, CIÊNCIA E TECNOLOGIA DO AMAPÁ, Campus Agrícola Porto Grande, Rodovia BR 210, Km 103, bairro Zona Rural, Porto Grande, AP Brasil

**Keywords:** *Phaseolus vulgaris* L., Selection index, Simultaneous selection, Selection gains, Grain yield

## Abstract

Common bean provides diet rich in vitamins, fiber, minerals, and protein, which could contribute into food security of needy populations in many countries. Developing genotypes that associate favorable agronomic and grain quality traits in the common bean crop could increase the chances of adopting new cultivars black bean. In this context, the present study aimed at selection of superior black bean lines using multi-variate indexes, Smith-Hazel-index, and genotype by yield*trait biplot analysis. These trials were conducted in Campos dos Goytacazes - RJ, in 2020 and 2021. The experimental design used was randomized blocks, with 28 treatments and three replications. The experimental unit consisted of four rows 4.0 m long, spaced at 0.50 m apart, with a sowing density of 15 seeds per meter. The two central rows were used for the evaluations. The selection of superior genotypes was conducted using the multiple trait stability index (MTSI), multi-trait genotype-ideotype distance index (MGIDI), multi-trait index based on factor analysis and genotype-ideotype distance (FAI-BLUP), Smith-Hazel index, and Genotype by Yield*Trait Biplot (GYT). The multivariate indexes efficiently selected the best black bean genotypes, presenting desirable selection gains for most traits. The use of multivariate indexes and GYT enable the selection of early genotypes with higher grain yields. These lines G9, G13, G17, G23, and G27 were selected based on their performance for multiple traits closest to the ideotype and could be recommended as new varieties.

## Introduction

Common beans are rich in protein [1], with high nutritional quality, antioxidant and anti-inflammatory properties, and could help reduction of obesity and cardiovascular diseases [2–4]. Brazil is one of the world’s largest producers and consumers of common bean, with an average yield of 1,043 kg ha^− 1^, varying from one location to the other location [5]. Researchers strive to create genotypes with valuable trait combinations, but it’s challenging to combine many desirable traits [5]. Selecting superior genotypes is complex due to the quantitative nature of important agronomic traits [6].

In the final stage of bean cultivar development, breeders prioritize specific traits linked to grain yield, as selection based on multiple traits couldan disrupt trait balance and interact with the environment negatively. Selection indexes such as Smith-Hazel (SH) index are employed to stream line trait selection [7, 8]. While the SH index is commonly utilized as a multi-trait selection index, there is substantial evidence suggesting that its application may not be advantageous in plant breeding. This holds true not only in initial trials, as indicated by Bhering et al [9], but also in the more advanced phases of breeding programs, as observed by Jahufer and Casler [10]. These advanced stages often involve multi-environment trials, as demonstrated by Dallo’ et al [11], Olivoto et al [12], and Woyann et al [13].

Olivoto and Nardino [14] developed a new simultaneous selection index based on factor analysis, known as Multi-Trait Genotype-Ideotype Distance Index (MGIDI), to circumvent the adversities encountered in traditional indexes, focused on genotype selection and treatment recommendation based on multiple trait information. The proposed index’s effectiveness was assessed through Monte Carlo simulations. These simulations involve evaluating its performance in selecting traits with desired gains under various scenarios. These scenarios encompass a range of factors, such as the number of genotypes, the traits under consideration, and the correlation structure between these traits. Yan and Frégeau-Reid [15] introduced the GYT approach for selecting superior genotypes by considering multiple traits. In crops, grain yield is often the key trait, and GYT analysis helps to identify genotypes that excel in both yield and other important variables, not just individual traits [8, 13].

Given the context, the present study aimed to select superior black bean lines using the multi-trait genotype-ideotype distance index and Smith and Hazel index and to evaluate the genotypes with multiple traits utilizing the genotype by yield*trait biplot analysis.

## Materials and methods

### Cultivation site and experimental design

**Fig. 1:**
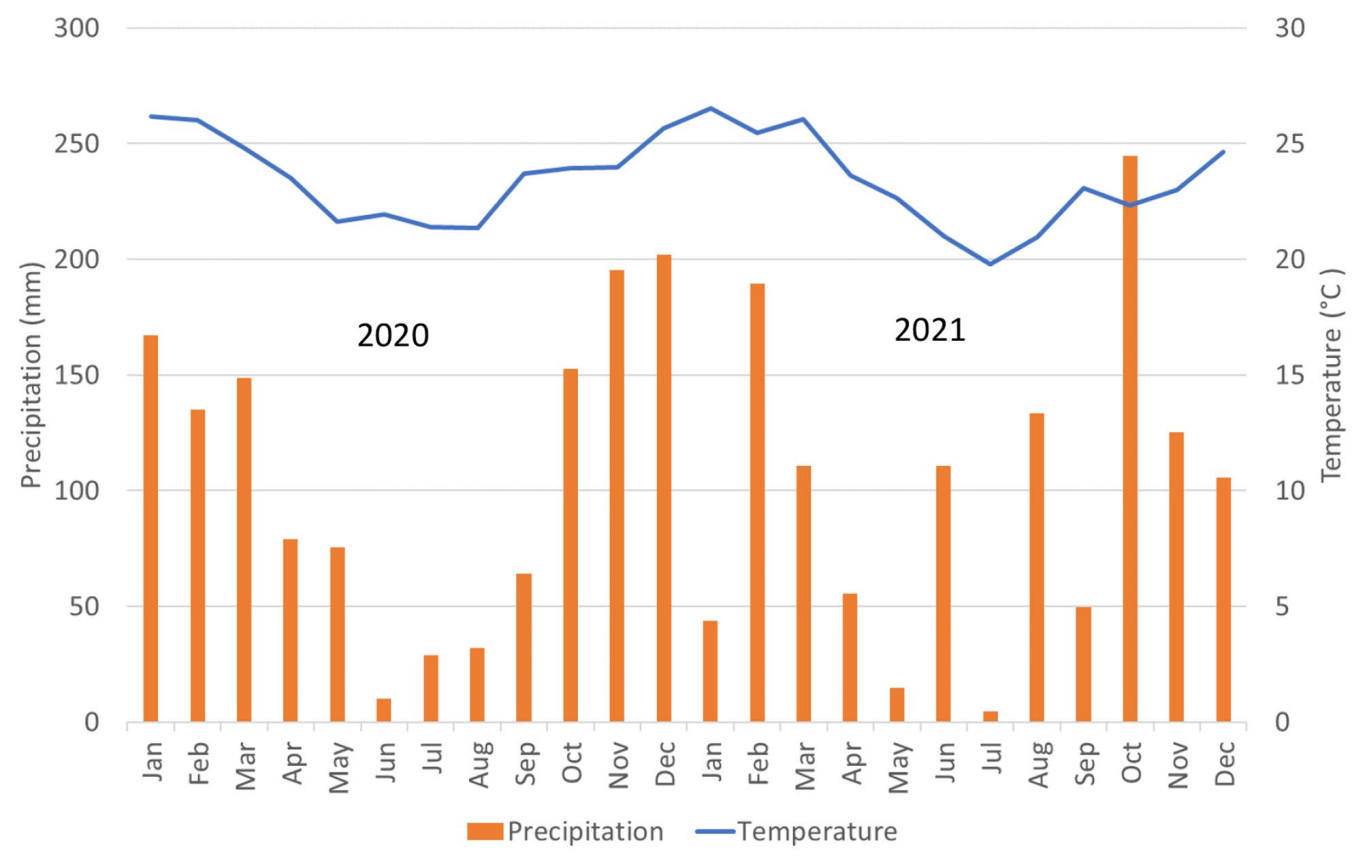
Temperatures and precipitation during the black bean experiment. Campos dos Goytacazes, Rio de Janeiro, 2020–2021

The trials were conducted in Campos dos Goytacazes -RJ, in 2020 and 2021. The municipality is located in the northern region of Rio de Janeiro, at 21º 19’ 23’’ S and 41º 19’ 40’’ W, with an altitude ranging from 20 to 30 m. According to the Köppen classification [16], the region’s climate is humid tropical (Aw), with rainy summers and dry winters. According to the Weather Station, the location has a small temperature range and average annual precipitation of 1,055.3 mm (Fig. 1).

A randomized block experimental design was used, with 28 genotypes (Table 1) and three replications. The experiment consisted of 28 genotypes of normal and early cycle black beans, of which 23 genotypes were advanced lines developed by Embrapa Arroz e Feijão and five control cultivars (BRS-ESTEIO, BRS-CAMPEIRO, BRS-FP403, IPR-UIRAPURU and IAC-VELOZ) The experimental plot consisted of four rows 4.0 m long, spaced at 0.50 m apart, with a sowing density of 15 seeds per meter. Only central rows were used for the evaluation and data recording on days to flowering (DF), pod length (PL), number of pods per plant (NPP), number of grains per pod (NGP), 100-grain mass (100 M), and grain yield (YIEL). The grain yield was evaluated by a manual harvest of the plants contained in the two central rows of 4.0 m in length in each experimental unit. The plants, after being uprooted, were dried in the sun and then mechanically threshed. The grains were weighed, the yield was estimated in kg ha^− 1^, and the humidity was corrected to 13%.

**Table 1:** Relationship of genotypes of normal black beans and early black beans, evaluated in the municipality of Campos dos Goytacazes in the State of Rio de Janeiro

ID	Genotype	Type	Source	ID	Genotype	Type	Source
G1	BRS-ESTEIO	N	Embrapa	G15	CNFP17494	N	Embrapa
G2	BRS-CAMPEIRO	P	Embrapa	G16	CNFP17466	P	Embrapa
G3	BRS-FP403	N	Embrapa	G17	CNFP19248	N	Embrapa
G4	IPR-UIRAPURU	N	IAPAR	G18	CNFP18310	P	Embrapa
G6	IAC-VELOZ	P	IAC	G19	CNFP19263	N	Embrapa
G7	CNFP16422	P	Embrapa	G20	CNFP19740	P	Embrapa
G7	CNPF16422	N	Embrapa	G21	CNFP19266	N	Embrapa
G8	CNFP17435	N	Embrapa	G22	CNFP19741	P	Embrapa
G9	CNFP17058	P	Embrapa	G23	CNFP19325	N	Embrapa
G10	CNFP17445	N	Embrapa	G24	CNFP19745	P	Embrapa
G11	CNFP17457	P	Embrapa	G25	CNFP19347	N	Embrapa
G12	CNFP17456	N	Embrapa	G26	CNFP19746	P	Embrapa
G13	CNFP17489	P	Embrapa	G27	CNFP19349	N	Embrapa
G14	CNFP17459	N	Embrapa	G28	CNFP19747	P	Embrapa

ID: Identification; N = Normal-cycle; P = Early-cycle. G: Genotype

### Statistical analysis

The restricted maximum likelihood/best linear unbiased prediction (REML/BLUP) approach was employed, utilizing the following model:

$$\text{y= }\text{Xb}\text{ + }\text{Zg}\text{ + }\text{Wc}\text{ + e}$$

y: Represents the data vector for fixed effects, which are block averages across different environments. b: Denotes the vector of fixed effect coefficients. g: Stands for genotype effects, which are considered random. c: Corresponds to genotype-environment interaction effects, also treated as random. e: Signifies random errors in the model. Additionally, the matrices X, Z, and W serve as incidence matrices for the fixed effects (b), genotype effects (g), and genotype-environment interaction effects (c), respectively.

### Multiple trait Stability Index (MTSI)

The process of choosing individuals with average performance and stability, taking into account multiple traits, relied on the assessment of genotype-ideotype distance. This distance was measured using Euclidean distance, utilizing scores derived from an exploratory factor analysis, outlined as follows

$$X=\mu +Lf+\epsilon$$

*X* represents a ×1*p*×1 vector of observations; *µ* is a 1*p*×1 vector of means; *L* is 1*p*×1 matrix of factor loadings; *f* is a 1*p*×1 vector of common factors; *ε* is a 1*p*×1 vector of residuals, where *p* and *f* denote the number of traits and retained common factors, respectively. The eigenvalues and eigenvectors were derived from the correlation matrix of *rXij*​. Initial loads were determined by considering only factors with eigen values greater than 1. Analytical rotation and estimation of final loads were accomplished using the varimax rotation criterion. Genotype scores were calculated based on the equation:

$$F=Z{\left({A}^{T}{R}^{-1}\right)}^{T}$$

*F* is a *g*×*f* matrix containing factorial scores; *Z* is a *g*×*p* matrix featuring standardized means; *A* is a *p*×*f* matrix representing canonical loads; *R* is a *p*×*p* correlation matrix among the traits. Here, *g*, *f*, and *p* denote the number of genotypes, retained factors, and analyzed traits, respectively.

According to the definition, the ideotype attains the maximum WAASBY score (100) for all variables under analysis. The calculation of WAASBY follows the given equation:

$$WAASB=\sum _{k=1}^{p}|{IPCA}_{ik}x{EP}_{k}|/\sum _{k=1}^{p}{EP}_{k}$$

In this context: WAASB represents the weighted average of absolute scores for the i-th genotype; IPCAik is the score of the i-th genotype along the k-th principal interaction component axis (IPCA); and EPk signifies the amount of variance explained by the k-th IPCA. It is noteworthy that, as established by Olivoto et al. [12], the genotype characterized by the lowest WAASB value is regarded as the most stable.

The last and third phase involved calculating the multi-trait stability index (MTSI) based on the provided equation:

$${MTSI}_{i}={\left[\sum _{j=1}^{f}{({F}_{ij}-{F}_{j})}^{2}\right]}^{\text{0,5}}$$

In this context: MTSIi represents the multi-trait stability index corresponding to the i-th genotype, Fij indicates the j-th score associated with the i-th genotype, and Fj denotes the j-th score linked to the ideotype. Genotypes exhibiting a reduced MTSI are in proximity to the ideotype, showcasing elevated performance and stability across all scrutinized variables. The calculation of the selection differential for mean performance was conducted for each trait, considering a selection intensity set at 30%.

### Genotype-ideotype multi-trait distance index (MGIDI)

The MGIDI distance index, introduced by Olivoto and Nardino [14] was applied to pinpoint the genotypes that effectively combine the majority of traits within each environment in a desired manner. MGIDI consists of knowing the optimal genotype and rescaling the variables so that they are all in a range of 0-10014, according to the following equation [14]:

$${rX}_{ij}=\frac{{\eta }_{nj}-{\phi }_{nj}}{{\eta }_{oj}-{\phi }_{oj}}x\left({\theta }_{ij}-{\eta }_{oj}\right)+{\eta }_{nj}$$

where *η*_*nj*_ and *φ*_*nj*_ are the new maximum and minimum values for trait *j* after rescaling, respectively; *η*_*oj*_ and *φ*_*oj*_ are the original maximum and minimum values for trait *j*, respectively, and *θ*_*ij*_ is the original value for the *j-th* trait of the *i-th* genotype. For DF in which lower values are desired, *η*_*nj*_ = 0 and *φ*_*nj*_ = 100 are considered. For all other traits where higher values are desired, *η*_*nj*_ = 100 and *φ*_*nj*_ = 0 were considered. Thus, the optimal genotype would be 100 for all traits after rescaling.

Subsequently, exploratory factor analysis was performed with *rXij* to group the related traits and reduce the dimensionality of the data, generating factor loads for each genotype [14] through the following equation:

$$X=\mu +Lf+\epsilon$$

*X* is a 1*p*×1 vector representing rescaled observations; *µ* is a 1*p*×1 vector denoting standardized means; *f* is a 1*p*×1 vector representing common factors; *ε* is a 1*p*×1 vector of residuals, where *p* and *f* are the numbers of retained traits and common factors, respectively. The eigenvalues and eigenvectors are derived from the correlation matrix of *rXij*. Only those with eigenvalues exceeding 1 are retained. The scores are computed using the equation:

$$F=Z{\left({A}^{T}{R}^{-1}\right)}^{T}$$

In this context: *F* is a *g*×*f* matrix containing factorial scores; *Z* is a *g*×*p* matrix featuring standardized means; *A* is a *p*×*f* matrix representing canonical loads; *R* is a *p*×*p* correlation matrix among the traits. Here, *g*, *f*, and *p* indicate the number of genotypes, retained factors, and analyzed traits, respectively. Subsequently, the computation of the Euclidean distance between genotype scores and ideal genotypes was conducted, determined by the MGIDI index [14] using the following equation:

$${MGIDI}_{i} = {\left[\sum _{j=1}^{f}{({Y}_{ij}-{Y}_{j})}^{2}\right]}^{\text{0,5}}$$

*Yij* denotes the score of the *i*-th genotype on the *j*-th factor (*i* = 1,2,…,*g*;*j* = 1,2,…,*f*), where *g* and *f* represent the number of genotypes and factors, respectively.*Yj* signifies the score of the *j*-th ideotype. The genotype with the minimum MGIDI is in proximity to the ideotype, showcasing desirable values for all the assessed traits. The selection differential for all traits was computed with a selection intensity set at 30%. Consequently, genotypes with lower MGIDI, i.e., those closer to the ideotype, were chosen.

### Multi-trait index based on factor analysis and genotype-ideotype distance (FAI-BLUP)

To obtain the FAI-BLUP, the distance between the ideotype and each genotype was estimated and then converted into a spatial probability to rank the genotypes [7]. The formula for calculating the FAI-BLUP index is as follows:

$${P}_{ij}=\frac{\frac{1}{{d}_{ij}}}{{\sum }_{i=1;j=1}^{i=n;j=m}\frac{1}{{d}_{ij}}}$$

where: *P*_*ij*_ is the probability that genotype (*i* = 1, 2,…,*n*) is similar to ideotype *j* (*j* = 1, 2,…,*m*); *d*_*ij*_ is the genotype to ideotype *j* distance, based on the standardized mean Euclidean distance.

### Smith-Hazel Index

The formula for calculating the Smith-Hazel index (SH) for the classic method of multi-trait stability assessment is as follows:

$${I}_{i}=\sum _{k}{b}_{k}{\stackrel{-}{y}}_{ik}$$

𝐼𝑖*Ii* represents the value of the index computed for progeny *i*; 𝑏𝑘*bk* is the weighting coefficient associated with trait *k*; *yik* signifies the phenotypic mean of progeny *i* in relation to trait *k*. The values of 𝑏𝑘*bk* were determined through *b* = *P*^− 1^*Gxa*, where:*P*^− 1^ is the inverse of the matrix representing mean phenotypic covariances between traits; *G* is the matrix indicating genotypic variances and covariances in the progeny mean across traits; *a* is the vector containing the economic weights of the traits. A selection intensity of 30% was employed.

### Genotype by Yield*Trait (GYT) biplot

The Genotype by Yield*Trait analysis employed the theoretical framework introduced by Yan and Frégeau-Reid [15]. This analytical approach relies on the utilization of phenotypic averages. When the breeder’s objective is to augment a particular variable, it is multiplied by the grain yield. Conversely, when the breeder aims to diminish a trait, such as, for instance, the number of days to flowering, the variable’s mean is divided by the grain yield. Prior to conducting the GYT analysis, the data underwent standardization to ensure that the mean for each trait was adjusted accordingly. The standardization process adhered to the following equation:

$${P}_{ij}=({T}_{ij}-\stackrel{-}{T}/{S}_{j})$$

In the context of this equation: ${P}_{ij}$ = represents the standardized value of genotype i for the specific variable or the combination of grain yield and trait *j*.; ${T}_{ij}$= signifies the original value of genotype i for the specific variable or the combination of grain yield and trait j in the GT or GYT table. $\stackrel{-}{T}$ = stands for the mean value across all genotypes for the specific variable or the combination of grain yield and trait j. ${S}_{j}$= represents the standard deviation for the specific variable or the combination of grain yield and trait j.

The GYT Biplot utilized the initial two principal components (PC) derived through singular value decomposition (SVD). Nevertheless, prior to conducting simultaneous selection, standardization was applied to all the data. The SVD conducted on the GYT table was subsequently transformed into genotype eigenvalues, trait eigenvalues, and singular values, following the equation introduced by Yan and Frégeau-Reid [15]:

$${P}_{ij}=\left({d\lambda }_{1}^{\alpha }{\xi }_{i1}\right)\left(\frac{{\lambda }_{1}^{1-a} {\tau }_{1j}}{d}\right)+\left({d\lambda }_{2}^{a} {\xi }_{i2}\right)\left(\frac{{\lambda }_{2}^{1-a} {\tau }_{2j}}{d}\right)+{\epsilon }_{ij}$$

where: *ξ*_*i1*_ and *ξ*_*i2*_ are the eigenvalues of PC1 and PC2 for genotype i, respectively; τ_1*j*_ and τ_2*j*_ are the eigenvalues of PC1 and PC2, respectively, for the combination grain yield x trait *j*; *λ1* and *λ2* are the singular values of PC1 and PC2, respectively; α is the singular value partitioning factor; *d* is the scalar distance that was chosen so that the longest length of the vector between the genotypes remained equal to the length between the traits; *ε*_*ij*_ is the residual of the fit of PC1 and PC2 for genotype *i* in the combination grain yield x trait *j*.

## Results

It is observed that the estimate of residual variance presented the highest proportion of the phenotypic variance for all variables. However, for days of flowering (DF), the proportion of genotypic variance estimate (1.07) was similar to estimates of residual variance (1.08), reflecting directly on higher heritability (Table 2).

The highest heritability estimates were observed for DF (0.50) and 100 M (0.42). The other variables showed low or moderate values for heritability, ranging from 0.08 for NPP to 0.19 for yield. Regarding the accuracy of the selection of genotypes, variations from 0.46 to 0.87 were observed for NPP and DF, respectively. The ratio between *CVg/CVr* ranged from 0.30 for NPP to 1.0 for the DF.

**Table 2:** Estimated variance components and genetic parameters for days to flowering (DF), pod length (PL), number of pods per plant (NPP), number of grains per pod (NGP), 100-grain mass (100 M), and grain yield (YIEL) evaluated in 28 black bean genotypes

Component	DF	PL	NPP	NGP	100 M	YIEL
${\widehat{\sigma }}_{g}$	1.07	0.05	3.60	1.96	2.81	11580.09
${\widehat{\sigma }}_{r}$	1.08	0.54	40.81	11.09	4.06	49532.12
${\widehat{\sigma }}_{f}$	2.15	0.60	44.41	13.05	6.87	61112.20
${h}^{2}$	0.50	0.09	0.08	0.15	0.42	0.19
*Accuracy*	0.87	0.48	0.46	0.59	0.54	0.64
*CVg*	2.24	1.91	12.48	17.65	4.60	10.72
*CVr*	2.25	6.04	42.04	42.01	12.57	22.17
*CVg/CVr*	1.00	0.32	0.30	0.42	0.37	0.48

### Selected genotypes and coincidence index

**Fig. 2:**
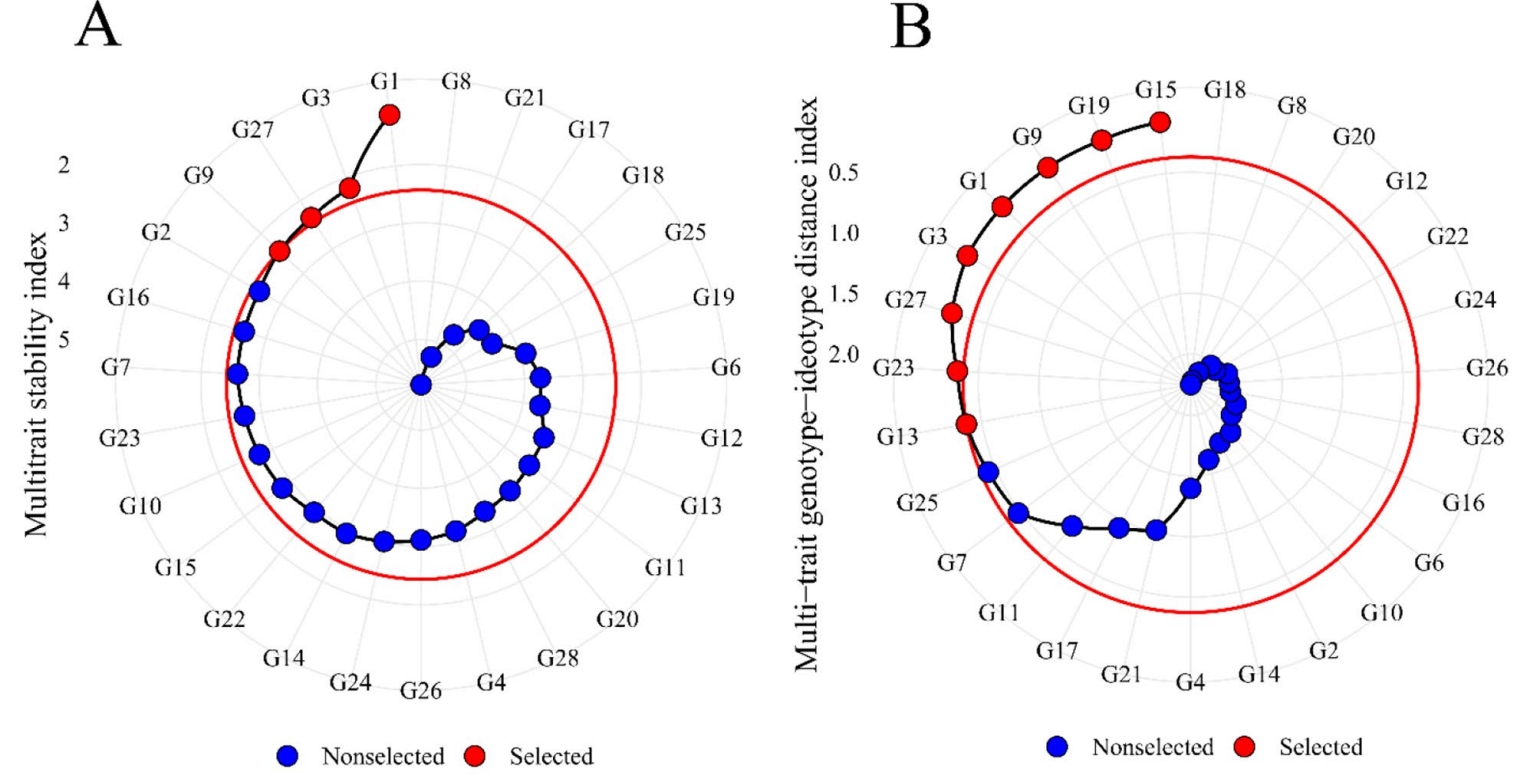
Multi-trait stability index (MTSI) (**A**) and Multi-trait genotype-ideotype distance index (MGIDI) (**B**) for 28 black bean genotypes. Selected genotypes are indicated in red, and the red circle represents the cut-off point according to the selection pressure

A 30% selection intensity was assumed for the selection indexes, represented by the red circle. It can be observed that the MTSI index selected only four genotypes, G1, G3, G27, and G9 (Fig. 2A). For the MGIDI index, eight genotypes were selected, G15, G19, G9, G1, G3, G27, G23, and G13 (Fig. 2B). The genotypes G2 and G25 were close to the cutoff point (red circle) for the MTSI and MGIDI indexes, respectively.

**Fig. 3:**
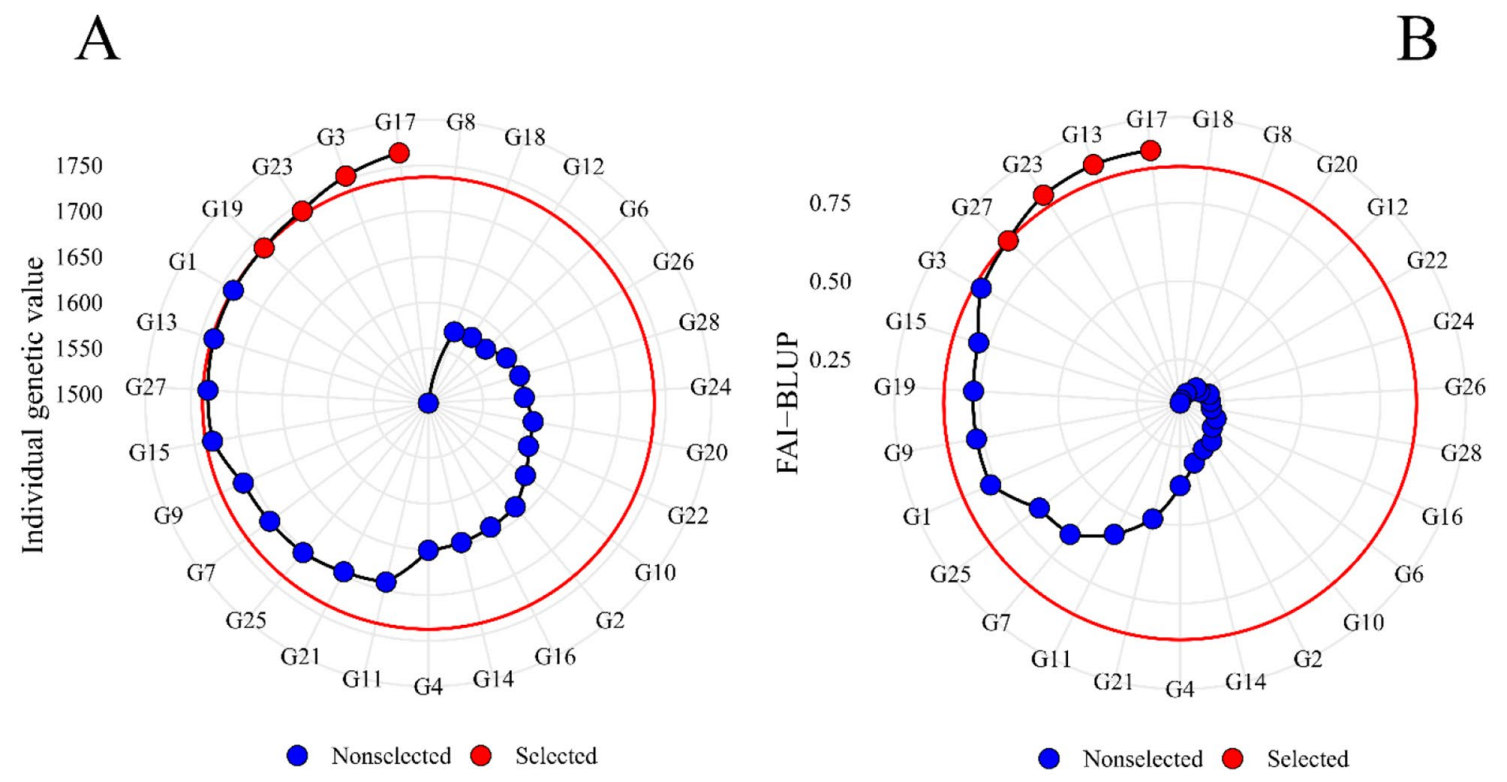
Smith-Hazel index (**A**) and FAI-BLUP index (**B**) for 28 black bean genotypes. Selected genotypes are indicated in red, and the red circle represents the cutoff point according to selection pressure

The Smith-Hazel (Fig. 3A) and FAI-BLUP (Fig. 3B) indexes selected four genotypes, G17, G3, G23, and G19 and G17, G13, G23, and G3, respectively. It is worth noting that both indexes selected the genotypes G17 and G23 in the same position.

**Fig. 4:**
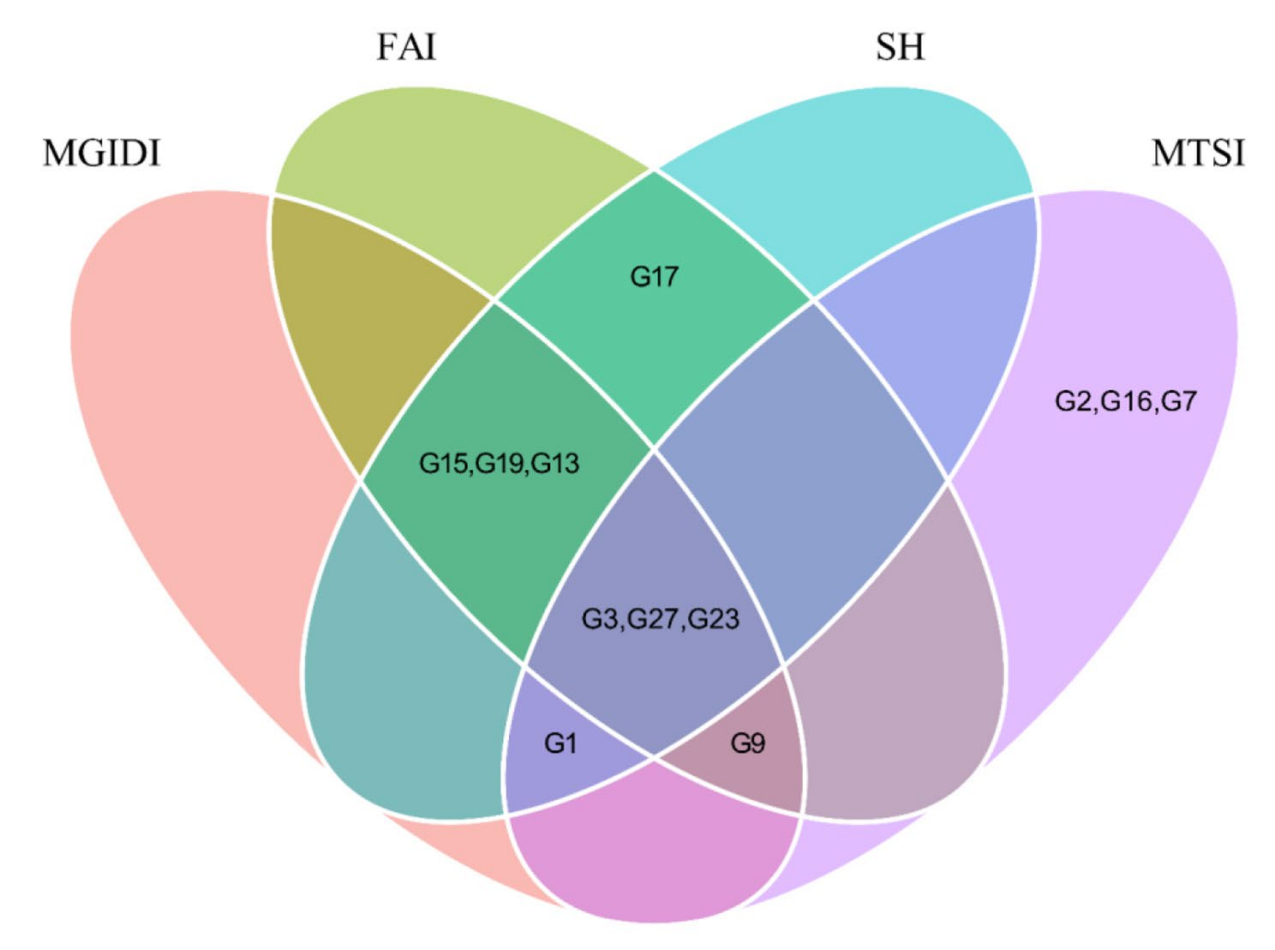
Venn diagram with the genotypes selected by the multi-traits stability index (MTSI), multi-trait genotype-ideotype distance index (MGIDI), Smith-Hazel index (SH), and FAI-BLUP index for 28 black bean genotypes

Genotypes G3, G27, and G23 were selected more often, followed by G1, G9, G15, G19, and G13 (Fig. 4), implying that these two genotypes performed better and were more stable in different environments. Of the eight genotypes selected, the MGIDI index shares the four selected by the FAI-BLUP index and SH index. The genotypes G3, G23, and G27 were common to all indexes; this suggests that these genotypes show a wide adaptation, performing well in different environments. Genotype G17 was selected exclusively by the FAI-BLUP index and SH. They were suggesting a close adaptation of this genotype.

It is observed that the variable 100 M obtained the lowest gain in the direct selection. However, it showed positive gains in the indirect selection indexes (Table 3). The NGP variable showed a higher frequency of negative selection deviation (SD) in all indexes, with FAI-BLUP indentation. This can cause a high frequency of selected genotypes with negative individual selection deviations. We should also consider that the selection objective of the DF variable is for reduction; thus, only direct selection and the FAI-BLUP index showed gains for this variable.

**Table 3:** Genetic selection differential based on direct selection (DS) and indirect selection via MTSI, MGIDI, SH, and FAI-BLUP indexes, considering selection intensity of 30%

Variable	Selection Differential (%)				
	SD	MTSI	MGIDI	SH	FAI-BLUP
DF	-0.38 (0.82)	0.04 (0.08)	0.50 (1.08)	0.80 (1.73)	-0.75 (1.63)
PL	0.28 (2.31)	0.02 (0.17)	0.10 (0.85)	0.15 (1.22)	-0.02 (0.14)
NPP	0.43 (2.81)	1.60 (10.47)	1.24 (8.13)	1.29 (8.47)	-0.93 (6.09)
NGP	0.31 (3.84)	-0.67 (8.48)	-0.90 (11.36)	-0.86 (10.8)	0.94 (11.90)
100 M	0.01 (0.06)	1.21 (6.15)	0.65 (3.30)	0.68 (3.45)	0.61 (3.10)
YIEL	14.65 (1.46)	114.80 (11.42)	84.05 (8.37)	88.20 (8.78)	93.20 (9.28)

DF - days to flowering, PL - pod length, NPP - number of pods per plant, NGP - number of grains per pod, 100 M − 100-grain mass, and YIEL - grain yield

Among the selected variables, YIEL, 100 M, and NPP showed the highest genetic gains (11.42%, 6.15%, and 10.47%) in the MTSI index. Regarding the FAI-BLUP index, it was the only one that showed negative gains for the variables PL and NPP, 0.14% and 6.09%, respectively. The MTSI index generally provided higher total gains, i.e., 28.3% for the variables with positive selection differential.

**Fig. 5:**
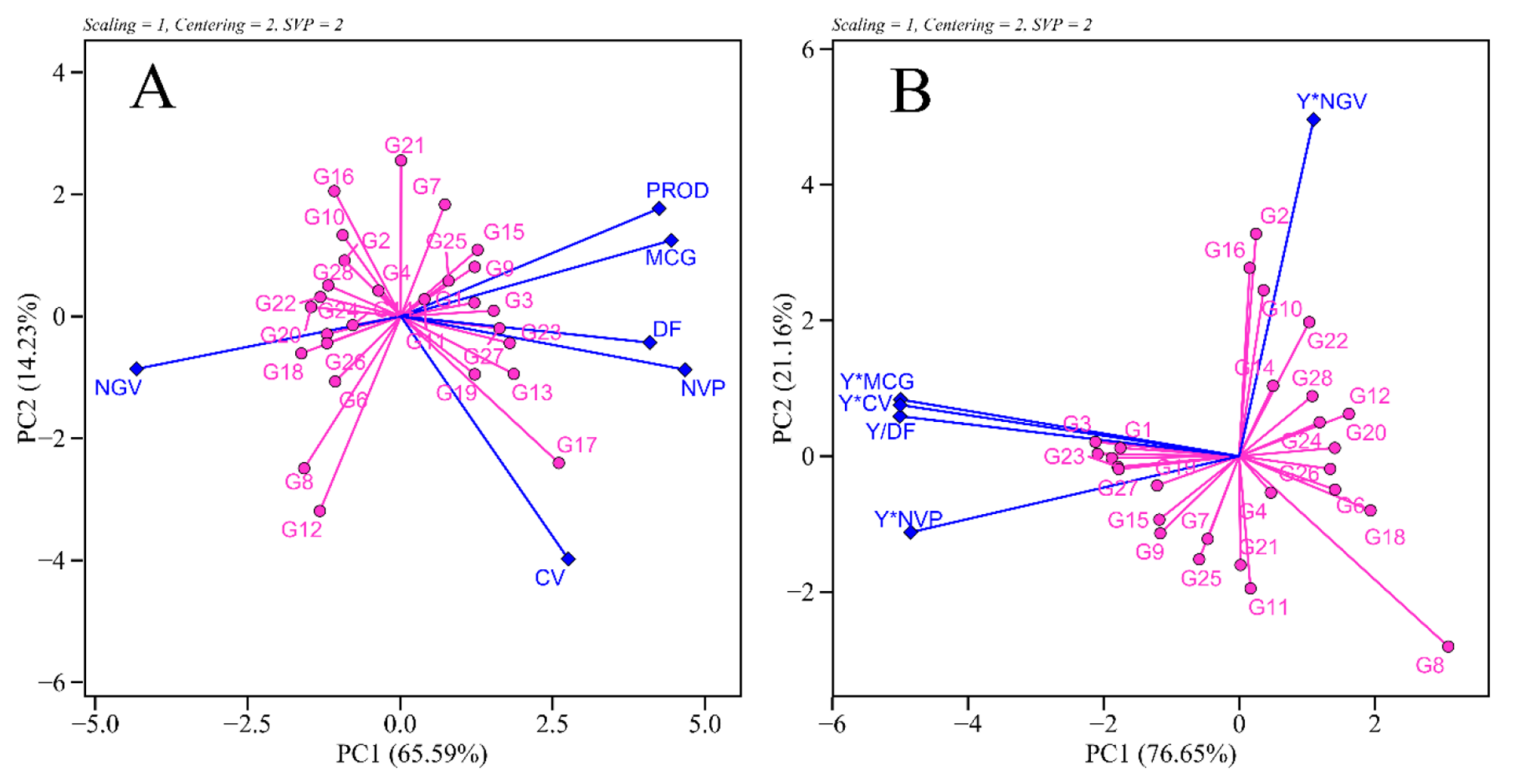
Genotype by trait biplot (**A**) and genotype by yield*trait biplot (**B**) of 28 black bean genotypes grown in 2020 and 2021

The GT-biplot analysis captured 79.82% of the variance, with PC1 contributing 65.59% and PC2 14.23% (Fig. 5A). Conversely, the genotype by yield*trait biplot exhibited PC1 (76.55%) and PC2 (21.16%), totaling 97.71% of the total variance explained by the first two axes (Fig. 5B). Both methods displayed sufficient explanatory power for data visualization, as more than 70% of the variance should be accounted for in graphical representations.

**Fig. 6:**
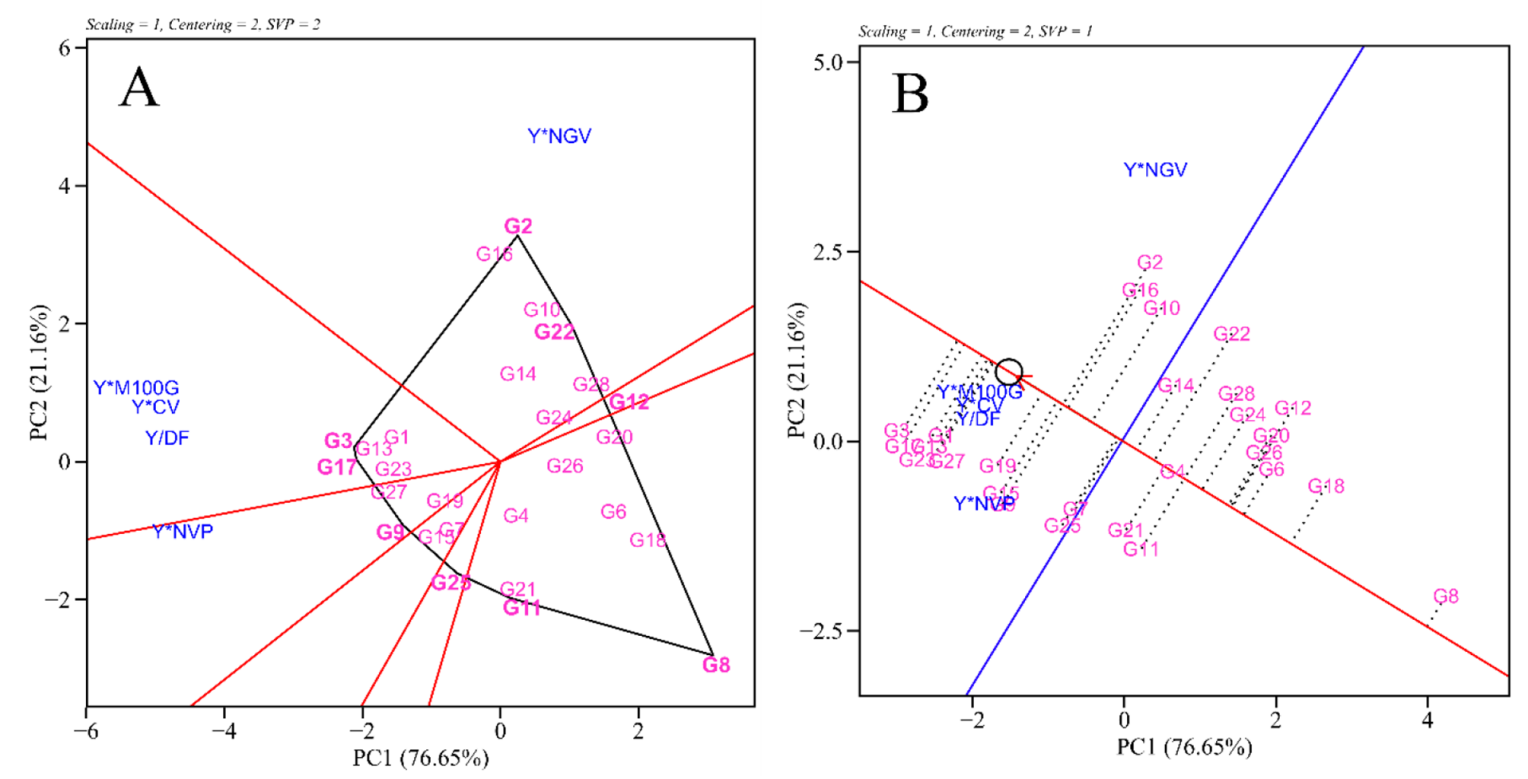
Who-wins-where biplot considering genotype by yield*trait (**A**) and mean, adaptability, and stability for genotype by yield*trait (**B**) of 28 black bean genotypes grown in 2020 and 2021

A polygon was formed regarding the who-wins-where biplot (Fig. 6A), derived from the linkage between genotypes characterized by the longest vertices in various directions, a polygon was delineated. Along each side of this polygon, a line was extended from the origin of the Biplot, represented by a red line, effectively partitioning it into sectors that portray the genotypic profiles across different traits. Within this framework, it became evident that the genotypes located at each vertex exhibited the most elevated values when considering the combinations of grain yield and traits.

The polygon showed the formation of seven sectors, but only three contained the variables analyzed. At the apex of the first sector, G2 showed the highest values for Y*NGP, and genotype G16 also showed potential for this combination. The genotypes G3 and G17 were the best for Y*100 M, Y*PL, and Y/DF combinations. The genotype G9 was superior in the Y*NPP combination.

Regarding the “means x stabilities” plot (Fig. 6B), it observes two lines intersecting at the origin of the biplot (center of the plot). The red line is the axis of the mean tester (MTA). The location of the MTA in the figure denotes the average placement of all yield*trait combination vectors. The arrow points to higher average values of the genotypes across all yield*trait combinations. The MTA serves to rank genotypes based on their overall superiority or utility. The blue line, meanwhile, separates genotypes with above-average overall performance from those with below-average overall performance [15].

The order of the genotypes that had superior rankings based on the ability to combine grain yield and target traits was G3 > G17 > G23 > G1 > G27 > G19. On the other hand, G8, G18, and G6 were considered the worst compared to the others. G3 and G17 were found to be the most balanced for several traits. Also, G1 is the most stable for most traits, however, it is within the group with overall below-average performance.

## Discussion

The *CVg/CVr* (Table 2) ratio estimates observed indicate that, in general, the variables analyzed show greater expression of the environmental component to the detriment of the genetic component and, consequently, lower gains with selection through these variables since they obtained moderate or low estimates of heritability (h^2^). As Cruz et al. [25] points out, the lower the genetic variance and the greater the environmental effect, the lower the heritability of the trait, which can be proved by the results obtained. The expression of these traits is complex within this scenario, given the large number of segregating locus controlling the trait, while suffering the influence of environmental effects. Consequently, understanding the heritability and the determining components of their variation are vital in the study of quantitative traits [26, 27].

For Resende [28], heritability is classified as low (h < 0.15), median (0.15 < h < 0.50), and high magnitude (h > 0.50). But it should be noted that values of small and medium magnitude for heritability are expected, mostly because it is a quantitative trait, which are susceptible to climate variations along the years. However, considering the values of the genetic coefficient of variation, which quantifies the magnitude of genetic variation available for selection, one can infer the existence of genotypes with superior genetic constitutions [29].

In advanced populations, genetic variability is lower than in the early stages. The probability of obtaining genotypes with simultaneously high individual selection differentials is lower than in early-stage populations subjected to higher selection intensity. This fact justifies the 30% selection intensity used in this study. However, the experimental design employed in this phase provides more accurate estimates of genotypic values due to replications and evaluations in multiple locations. In addition, the frequency of genotypes with undesirable traits that affect yield, such as susceptibility to pests and diseases, is lower.

The assessment of experimental precision was conducted by analyzing the estimates of selective accuracy. This particular parameter serves as an indicator of the effectiveness of both the information and methodologies employed for predicting genetic values. Selective accuracy, closely linked to the accuracy of selection, quantifies the correlation between the predicted genetic values and the actual genetic values of individuals, as described by Pimentel et al. [17]. According to Resende et al. [30], moderate to high accuracy values were observed, representing good accuracy in identifying superior individuals. States accuracy above 90% is only possible for traits with high heritability, and that accuracy values greater than 0.70 are sufficient to provide a more accurate inference about the genetic value of progenies. As a measurement related to precision in selection, accuracy is the main element of genetic progress which may be modified by a person aiming at maximizing genetic gain [30]. Most of the genotypes selected by the indexes were the advanced lines (Figs. 2 and 3). Thus, it can be inferred that the selected lines have the genetic potential to originate new cultivars, presenting the desired traits and, thus, differentiating themselves from cultivars already on the market. One should note, in addition to the selected individuals, those close to the cutoff point (red circle), which suggests that these genotypes may have interesting traits. Thus, the researcher should investigate genotypes very close to the cutoff point [12]. In a study with the species *Avena sativa L*., the authors Olivoto et al. [12] applied a selection intensity of 15%, the three selected genotypes were also within the cutoff point (red circle) considering the selection intensity. Therefore, in future studies it would be interesting to investigate the performance of genotypes that are very close to the cutoff point.

In the Veen diagram (Fig. 4), it is possible to verify that the common genotypes that were selected by all indices show broad adaptation, presenting good performance in different environments. Correlated data are common in breeding experiments [14]. The simulation study created by Olivoto et al. [12, 14] revealed that the pattern of correlation between characteristics will influence the success of the selection.The selection differentials for pod length were low for most indexes (Table 3). The not so expressive gains can be explained by the fact that simultaneous selection for several traits reduces the genetic gain per trait individually. However, for the traits where the gains were more reduced, Zetouni et al. [18] point out that the genetic gains in the trait set can balance this reduction [19]. In this study it is possible to apply the selection of superior genotypes through positive selection differentials for traits that wanted to increase and negative selection differentials for a trait that wanted to decrease. This should be useful for breeders and agronomists who aim at simultaneous selection for average performance and considering several traits, as it provides a unique selection process that is easy to interpret and considers the correlation structure between traits [12].

Overall, the MGIDI (Table 3) index provided satisfactory gains, with good effectiveness in selecting genotypes close to the ideotype where larger pod size, a higher number of pods per plant, 100-grain mass, and higher grain yield values are desired. In the simulation study on a simulated data set to evaluate the performance of the MGIDI index and compare it with the classic Smith-Hazel (SH) index and the modern FAI-BLUP index in terms of percentage of success in feature selection with desired gains, the MGIDI was found to outperform the FAI-BLUP and SH indexes in all simulation scenarios, and its superiority is more evident in datasets with a low correlation between traits. Generally, MGIDI presents 71.7% of success in selecting traits with desired gains [14]. The indexes’ performance is dependent on the number of traits, the number of genotypes analyzed and the degree of correlation between traits. The differences in selection success are more evident in datasets with a low correlation between traits, where the MGIDI index presents the higher success rates [14].

However, the gains obtained in these traits are accompanied by a reduction in the number of grains per pod. Negative selection differentials are interesting when aiming to reduce the variable in the ideotype design. That said, considering the FAI-BLUP index, we verified the possibility of success in using the selection index to reduce the cycle by reducing days to flowering and increasing the grain yield.

Comparing the Smith-Hazel index with the FAI-BLUP index, MTSI, and MGIDI, the sum of the SH selection differentials shows positive gains (23.56%). The total SH gains were close to those found for the MGIDI index when considering grain yield and yield components. However, the SH index was lower than that observed for MTSI and FAI-BLUP.

An ideotype-based breeding program focuses on multi-traits simultaneously. This method differs from other multivariate approaches in plant breeding, such as the Smith-Hazel index, which tends to focus on a few traits. Focusing directly on a few variables statistically simplifies the problem [20]; however, important information may be overlooked in data analyses.

Thus, several studies have reported the efficiency of multivariate selection indexes for simultaneous selection in plant breeding. Here are some instances: the identification of drought and salinity-resistant soybean genotypes [21], the development of bread wheat ideotypes tailored for early sowing conditions [22], the selection of millet strains with resistance against shoot fly infestations [23], and the breeding of chickpea genotypes with enhanced drought tolerance [24].

In the GYT-biplot analysis (Fig. 5), the variables tend to be positively correlated as they are yield components, even if these variables per se are negatively correlated [15]. This approach allows us to rank genotypes based on their levels of yield-trait combinations.

For Fig. 6A, it can be inferred that the genotypes G3 and G17 were the best for combining grain yield traits with grain mass and earliness. In bean breeding programs, cultivars are not selected only when they present a high grain yield. Other traits, such as DF and 100 M, are important to improve the quality and, consequently, the final value of the product. As a result, the earliness and grain size traits are relevant in the analyses for the ideotype of the bean. Thus, seeking to increase the variability, it would be interesting to cross these genotypes to obtain segregating populations with greater yield potential and precocity.

This superiority could be explained by the G3 cultivar (RS-FP403), which is already sold in the market for its superior agronomic traits, besides its ability to adapt to the conditions of the Cerrado and Atlantic Forest. The same interpretation can be made for the G1 genotype (BRS-Esteio), as it was among the genotypes with superior performance for all traits.

It should also be noted that ten genotypes, including the control cultivar, were present in those sectors that did not contain any yield*trait combinations, which implies that these genotypes obtained the worst performance of the traits studied in combination with grain yield compared to the rest of the genotypes.

The visualization of the mean tester axis (MTA) in the “means*stabilities” plot (Fig. 6B) is the unique trait of the GYT biplot, as it displays the rankings of competing genotypes based on the strengths and weaknesses of each genotype that cannot be visualized in other biplots, including GT biplot [24]. This view categorized the lower and upper genotype groups separated by the blue line.

In this study, the MTA biplot grouped 12 genotypes as superior. Among these genotypes, G3, G17, G23, and G1 had superior trait profiles, i.e., closer to the ideotype. It should be noted that these genotypes were the ones selected by the multivariate indexes, equally by the SH index, indicating the superiority of these genotypes and the coincidence of the indexes with the GYT biplot method in selecting superior genotypes.

Another important point is that the genotypes near the cutoff point (Figs. 2 and 3) are present among the group of superior genotypes (Fig. 6B), ratifying the importance of attention to this group of individuals, as they may present traits of interest. In contrast, the other inferior genotypes had an unfavorable trait profile when evaluated with grain yield, so that they can be rejected based on these multiple traits.

In situations where a breeding program aims to enhance the performance of multiple traits, tools akin to the GYT-biplot become pivotal. These tools enable the identification of genotypes that exhibit superior performance across multiple traits concurrently. The synergy between GYT-biplot analyses and multi-trait selection indexes, along with the incorporation of BLUP values, offers a significant advantage, as noted by Woyann et al. [13].

This is related to the fact that GYT-biplot analysis and multi-trait indexes stand out as useful methodologies that surpass classical methodologies to deal with the multi-trait scenario and by being a visual tool to describe the performance of genotypes and rank them. Ultimately, combining these analyses improves the reliability of the results.

## Conclusion

The multivariate indexes efficiently selected superior black bean genotypes, showing desirable selection gains for most traits.

The use of multivariate indexes and GYT enable the selection of early genotypes with higher grain yields. The lines G9 (CNFP17058), G13 (CNFP17489), G17 (CNFP19248), G23 (CNFP19325), and G27 (CNFP19349) were selected based on the best performance for multiple traits, are the closest to the ideotype, and can be recommended as new cultivars.

The phenotypic means of the selected lines were equal or higher when compared to the phenotypic means of the controls (control cultivars). This indicates the productive potential of the lines studied here, allowing them to be registered and recommended as new cultivars.

## Data Availability

The datasets generated and/or analysed during the current study are not publicly available because they will be used to be used for development of the black bean cultivars but are available from the corresponding author on reasonable request.
